# Potential utility of natural products as regulators of breast cancer-associated aromatase promoters

**DOI:** 10.1186/1477-7827-9-91

**Published:** 2011-06-21

**Authors:** Shabana I Khan, Jianping Zhao, Ikhlas A Khan, Larry A Walker, Asok K Dasmahapatra

**Affiliations:** 1 National Center for Natural Products Research, Research Institute of Pharmaceutical Sciences, School of Pharmacy, University of Mississippi, University, MS 38677, USA; 2 Department of Pharmacognosy, University of Mississippi, University, MS 38677, USA; 3 Department of Pharmacology, University of Mississippi, MS 38677, USA; 4 University of Mississippi Cancer Institute, University of Mississippi, University, MS 38677, USA

## Abstract

Aromatase, the key enzyme in estrogen biosynthesis, converts androstenedione to estrone and testosterone to estradiol. The enzyme is expressed in various tissues such as ovary, placenta, bone, brain, skin, and adipose tissue. Aromatase enzyme is encoded by a single gene *CYP 19A1 *and its expression is controlled by tissue-specific promoters. Aromatase mRNA is primarily transcribed from promoter I.4 in normal breast tissue and physiological levels of aromatase are found in breast adipose stromal fibroblasts. Under the conditions of breast cancer, as a result of the activation of a distinct set of aromatase promoters (I.3, II, and I.7) aromatase expression is enhanced leading to local overproduction of estrogen that promotes breast cancer. Aromatase is considered as a potential target for endocrine treatment of breast cancer but due to nonspecific reduction of aromatase activity in other tissues, aromatase inhibitors (AIs) are associated with undesirable side effects such as bone loss, and abnormal lipid metabolism. Inhibition of aromatase expression by inactivating breast tumor-specific aromatase promoters can selectively block estrogen production at the tumor site. Although several synthetic chemical compounds and nuclear receptor ligands are known to inhibit the activity of the tumor-specific aromatase promoters, further development of more specific and efficacious drugs without adverse effects is still warranted. Plants are rich in chemopreventive agents that have a great potential to be used in chemotherapy for hormone dependent breast cancer which could serve as a source for natural AIs. In this brief review, we summarize the studies on phytochemicals such as biochanin A, genistein, quercetin, isoliquiritigenin, resveratrol, and grape seed extracts related to their effect on the activation of breast cancer-associated aromatase promoters and discuss their aromatase inhibitory potential to be used as safer chemotherapeutic agents for specific hormone-dependent breast cancer.

## Background

**Figure 1:**
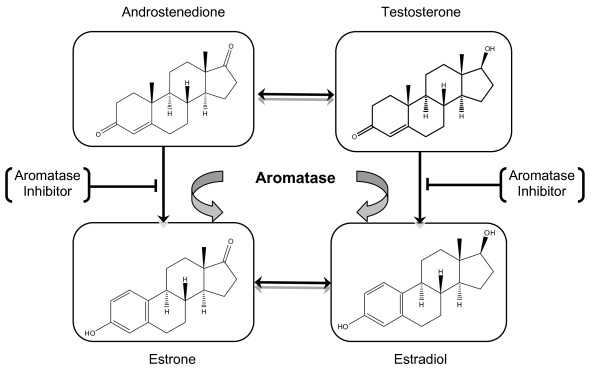
**Schematic diagram of the reaction catalyzed by aromatase enzyme**.

Aromatase is a member of the cytochrome P450 enzyme family and a product of the *CYP 19A1 *gene [1]. This membrane-bound protein (aromatase) is the rate limiting enzyme in the conversion of androstenedione to estrone (E1) and of testosterone to estradiol (E2) (Figure 1). Aromatase consists of two components: the hemoprotein aromatase cytochrome P450 encoded by the *CYP19A1 *gene and expressed only in steroidogenic cells, and the flavoprotein NADPH-cytochrome P450 reductase, expressed ubiquitously in many cell types [2-4]. The enzyme (aromatase) is localized in the endoplasmic reticulum of a cell, and catalyzes three hydroxylation reactions that convert androstenedione to E1 and testosterone to E2 [5,6]. The enzyme activity is increased by alcohol, age, obesity, insulin and gonadotropins [7]. The *CYP19A1 *gene is highly expressed in the human placenta and in the granulosa cells of the ovarian follicles. However, many nonglandular tissues including liver, muscle, brain, bone, cartilage, blood vessels, breast (both normal and carcinogenic) and adipose tissues have lower level of *CYP 19A1 *expression under the control of tissue-specific promoters [8]. Inhibition of aromatase enzyme activity has been shown to reduce estrogen production throughout the body and aromatase inhibitors (AIs) are being used clinically to retard the development and progression of hormone-responsive breast cancer [6,7].

## The aromatase gene and tissue-specific promoter expression

**Figure 2:**
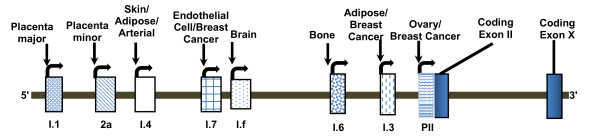
**Partial structure of human CYP19 gene**. Human aromatase gene is located on chromosome 15 and transcribes from telomere towards centromere. The aromatase gene is ~ 123 kb long contains nine coding exons (II-X) and two alternative polyadenylation sites. Partially tissue specific promoters direct aromatase gene transcription.

Human aromatase is a 58 kDa protein which was first purified from placental microsomes in 1980s [9]. Only recently has the crystal structure of human placental aromatase been described [5]. Aromatase is encoded by a single copy of the *CYP19A1 *gene which is ~123 kb long, located on the short arm of the chromosome 15 (15q21), and is transcribed from the telomere to the centromere [2,10-12]. The coding region spans 30 kb and includes nine translated exons (II-X) with two alternative polyadenylation sites [2]. The ATG translation initiation site is located on the exon II. There are a number of alternative non-coding first exons (I.1, I.2, I.3, I.4, I.5, I.6, I.7, and PII) which are expressed in tissue-specific manner, lie upstream to the coding region and are spliced to a common acceptor sites in exon 2 [13-15] (Figure 2). The distal promoter I.1 which drives transcription in placenta is located approximately 89 kb upstream of exon II. The proximal promoter found immediately upstream of exon II is PII which is expressed in the gonad. In between these two promoters, several other first exons and promoters have been identified, such as 2a in the placental minor, I.3 in the adipose tissue specific promoter, I.4 in the promoters in skin fibroblast and preadipocytes, I.5 in fetal, I.6 in bone, I.f in brain, and I.7 in endothelial cells [2,14,16-18]. As various tissues utilize their own promoters and associated enhancers and suppressors, the tissue-specific regulation of estrogen synthesis is very complex. Due to the use of alternative promoters, aromatase transcripts in various expression sites contain unique 5'-untranslated first exons, which are spliced onto the coding exon II at the common 3'-splice site upstream of the ATG translation start codon [14]. Although expression of the aromatase gene is under the control of distinct tissue-specific promoters, the coding region of aromatase transcripts and the resulting protein is identical in all expression sites [9,14] and [19].

In healthy breast tissue, expression of *CYP 19 *is under the control of promoter I.4 with synergistic actions of class I cytokines such as IL-6, IL-11, tumor necrosis factor-α (TNF-α) and glucocorticoids [9,20-22]. In tumorous tissue aromatase expression is switched to promoters I.3 and II which are transactivated by protein kinase A (PKA) and cAMP-dependent signaling pathways [8,23]. Depending on the microenvironment the promoter switching in the malignant breast tissue results in the enhancement of aromatase gene transcription, protein expression, and the enzymatic activity compared to the normal breast tissue. Moreover, this promoter switching is the primary reason for the increased estrogen production in adipose stromal cells surrounding the breast cancer [18,24,25]. Promoter I.7 is also considered to be a novel breast cancer associated aromatase promoter situated midway between promoter I.4 and promoter I.3/II [14,22]. Promoters I.3 and II lie 215 bp apart from each other and are coordinately regulated by prostaglandin E2 (PGE2) via a cAMP-PKA-dependent pathway, and not by cytokines as seen in normal breast tissue [8]. Signaling effects/transcriptional regulators that mediate PGE2 action include the activator pathways p38/CREB-ATF and JNK/jun and the inhibitory factor breast cancer 1 (BRCA1) in breast adipose fibroblasts [14,22].

## Breast cancer and aromatase

Breast cancer is an important public health problem worldwide. In the United States, breast cancer represents the most common neoplasm and second most frequent cause of cancer death in women [26]. Estrogens have been implicated in the etiology of breast cancer and have been added to the list of known human carcinogens [27,28]. Estrogens are suggested to cause breast cancer by stimulating cell growth and proliferation through receptor-mediated processes and via their genotoxic metabolites [29,30]; therefore, inhibition of estrogen production/effect is nowadays a common practice for breast cancer treatment [9]. The general strategies to inhibit estrogen action are to block estrogen receptor (ER) binding to its specific ligand or to disrupt estrogen production by altering the aromatase gene expression or enzyme activities [15]. ER antagonists can block estrogenic actions; however, estrogen production can be inhibited by aromatase inhibitors (AI).

It is very important to know that the E2 production site in women changes with the increase of age [6]. In the pre-menopausal period the ovaries are the major source of aromatase and its substrate, androstenedione and thus E2. In humans, androstenedione is produced by the theca folliculi cells, and is converted to E1 and subsequently to E2 in the granulosa cells by aromatase. Therefore, during the reproductive years, E2 mainly works as an endocrine factor acting on estrogen-sensitive tissues. In the post-menopausal period the ovaries lose the expression of aromatase; however, they are still able to produce androstenedione. At this stage adrenal glands are the major producer of androgens, which are converted to estrogens in peripheral tissues such as liver, fat, muscle, skin, bone, and mammary tissue [6,31-33]. In post-menopausal women E2 synthesized in extragonadal sites acts locally at these peripheral sites as intracrine, autocrine, paracrine or juxtacrine factors, and acts directly in the cells that synthesize estrogen or on the neighboring cells [34,35]. Moreover, in post-menopausal breast cancer patients, the concentration of E2 in breast tissue is ~20-fold greater than in plasma, suggesting that intratumoral estrogen synthesis, its retention, and cellular uptake plays important role in the progression of ER+ breast cancer [6,36]. Although the exact localization of aromatase in human breast tumor is still controversial [37,38], in majority of the breast cancer cases aromatase activity and aromatase mRNA levels show higher levels than those observed in non-malignant mammary tissues [39]; this supports the concept that in-situ production of E2 by aromatase plays a major role in breast cancer progression [40].

Considering the importance of E2 in hormone receptor positive breast cancer, many therapeutic approaches have been developed to deprive E2 signaling [7,9,15]. Two main chemical approaches have been successfully utilized [15]. The traditional method of E2 inhibition is to interfere with E2 interaction with its receptors (ERα and ERβ) using selective estrogen receptor modulators (SERMs) such as tamoxifen and raloxifene [41,42]. Another approach is to reduce E2 signaling by using AIs to decrease E2 synthesis [43]. While SERMs are effective both in pre-and post-menopausal women, AIs are not appropriate to use for pre-menopausal women, because in pre-menopausal women, AIs, by lowering the E2 levels, stimulate the secretion of gonadotropins from the pituitary gland. Subsequently, the gonadotropins stimulate the ovaries to produce estrogens which can counteract AIs effect and possibly causing ovarian cysts [44]. Moreover, due to indiscriminate reduction of aromatase activity in all expression sites of the body, AIs can induce many side effects such as bone loss, hepatic steatosis and abnormal lipid metabolism [14,45-49]. Therefore it is desirable to design selective aromatase modulators that target the over-expression of this enzyme (aromatase) in breast epithelial cells and surrounding fibroblasts, while other sites of estrogen production remained unaltered [50,51]. With this regard, selective inhibition of aromatase promoter I.3/II activities may be a fruitful approach to inhibit estrogen production in breast tumor while allowing aromatase expression via alternative promoters in other regions of the body like brain and bone.

## Inhibitory agents of aromatase promoter I.3/II

There are several potential synthetic agents available for inactivation of aromatase promoter I.3/II. Studies in human breast adipose fibroblasts revealed that sodium butyrate, peroxisome proliferator activated receptor γ (PPAR γ) agonists, retinoid X-receptor (RXR) agonists, and inhibitors of p38 and JNK are capable of inhibiting aromatase promoter I.3/II activity. The action of these agents has been summarized in a recent review by Chen et al [14]. However, these synthetic products are also known to induce side effects. Troglitazone, rosiglitazone and pioglitazone are PPARγ agonists (FDA approved rosiglitazone and pioglitazone for the treatment of type 2 diabetes). These drugs caused edema, reduced hemoglobin and hematocrit levels, increased plasma LDL-and HDL cholesterol and increased body weight [52-55]. The RXR agonist LG101305 (the FDA approved drug is bexarotene) induced hypertriglyceridemia, hypercholesterolemia, hypothyroidism and leucopenia. Sodium butyrate induced bradycardia [55-57] while p38 inhibitor SB202190 is toxic to liver and the JNK inhibitor AS601245 have no reported side effects compared to others [58-60].

## Natural products targeting aromatase gene promoters

With the clinical success of several synthetic AIs in the treatment of postmenopausal ER-positive breast cancer, researchers have also been focused onto the potential of natural products as AIs [61]. These compounds (natural products) are mostly obtained from terrestrial and marine organisms and are still in the forefront of drug discovery. Moreover, the rich structural diversity and complexity of these compounds prompted the researchers to synthesize them in the laboratory for therapeutic applications. Many chemopreventive drugs used today are derived from the natural products [62-68]. In addition, many natural products that have been used traditionally for nutritional or medicinal purposes as botanical dietary supplements (BDS) may also afford as AIs with reduced side effects [61,69,70]. Because many natural products are associated with low toxicity, they are potentially excellent candidates for use as chemopreventive agents [71-73]. Epidemiological evidence suggests that women living in Asia, where diets have traditionally included soybean products, report fewer postmenopausal symptoms and experience fewer breast cancers than women in Western countries [74-77]. More specifically, Asian women have a 3-fold lower breast cancer risk than women in the United States, independent of body weight [78]. Furthermore, serum concentrations of E2 are 40% lower in Asian women compared with their Caucasian counterparts [79]. Thus, environmental and dietary factors may explain at least some of the discrepancy in breast cancer risk between Asian and western populations [74,75]. Despite the known AIs, there is still a need of searching for new AIs from natural products for future drug development [68]

**Figure 3:**
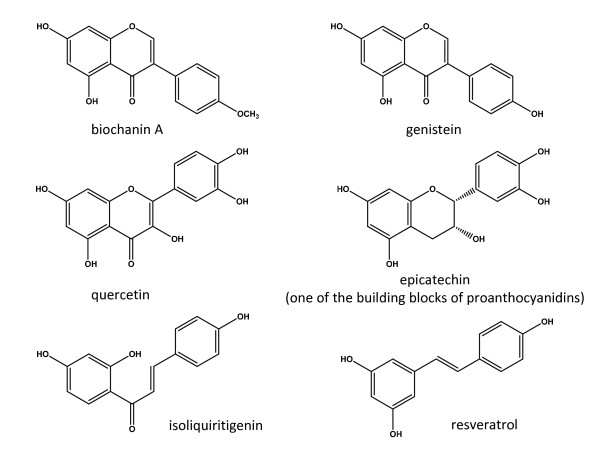
**The chemical structures of biochanin A, genistein, quercetin, epicatechin, isoliquiritigenin, and resveratrol**.

Among the natural products tested as AIs, phytoestrogens, such as flavones and isoflavones are able to bind ER and induce estrogen action [77]. The binding characteristics and the structural requirements necessary for the inhibition of human aromatase by flavones and isoflavones were obtained by using computer modeling and confirmed by site-directed mutagenesis [80-82]. It was found that these compounds bind to the active site of aromatase in an orientation in which their rings A and C mimic rings D and C of the androgen substrate, respectively [80]. Until now ~ 300 natural products, most of them are phytoestrogens, have been evaluated for their ability to inhibit aromatase using noncellular (mostly using human microsome as a source of aromatase enzyme), cell-based, and in vivo aromatase inhibition assays [61,83-85]; however, only a few studies (biochanin A from red clover, genistein from soybean, quercetin, isoliquiritigenin from licorice, resveratrol from grape peel and extracts of grape seeds, Figure 3) have been reported for their effect on aromatase promoter I.4, I.3/II activity [86-91]. The exact mechanisms how these plant products adapted to inhibit aromatase gene expression or enzyme activity is not fully understood.

Biochanin A (5, 7-dihydroxy-4'-methoxyisoflavone) is an isoflavone extracted from red clover (*Trifolium pretense*) by Pope et al. [92]. The first evidence that red clover has estrogenic activity were reported by Bennets et al. [93] after observing breeding problems of sheep grazing on red clover pastures which have been attributed to the isoflavone and coumestrol content of red clover. Serious fertility disturbances indicating estrogenic stimulation of cattle fed with red clover silage were reported [94-96]. Although biochanin A was moderately active in inhibiting microsomal aromatase activity (IC_50_: 5-10 μM) but was strongly active when tested in JEG-3 cells (human placental choriocarcinoma cell line). However, it did not inhibit aromatase activity in granulosa-luteal cells, and human preadipocyte cells and was also inactive in trout ovarian aromatase assay [61]. Interestingly, in MCF-7 cells (ER-positive breast cancer cells) biochanin A exhibited a dual action. It inhibited aromatase activity at low concentrations, but was estrogenic at high concentrations [97]. Furthermore, in SK-BR3 cells (ER-negative breast cancer cells) biochanin A was reported to inhibit aromatase enzyme activity and reduce mRNA expression. By using a luciferase reporter gene assay it was demonstrated that this phytochemical (biochanin A) was able to suppress the activation of breast-specific promoter I.3/II [88]. However, it is not known whether this inhibition is mediated through a PGE-2 or cAMP dependent PKA mechanisms. When genistein (a major metabolite of biochanin A) was tested in the same model, it was also found to suppress promoter I.3/II activation and showed an inhibition of aromatase enzyme activity [88]. Therefore, the inhibitory effect of biochanin A on aromatase promoter activation was suggested by the authors to be due to its metabolic conversion to genistein rather than its direct effect [88].

Genistein is a major phytoestrogen isolated from soybean, a potential nutraceutical, geared for women suffering from perimenopausal symptoms [98-101]. Genistein is also found in a number of other plants such as fava beans, lupin, kudzu, and psoralea [102]. Genistein is believed to be a chemopreventive agent against various types of cancers, including prostate, cervix, brain, breast, esophagus and colon [103]. Genistein was shown to increase aromatase activity in human adrenocortical carcinoma (H295R) cells and in isolated rat ovarian follicles [104,105]. Dietary genistein, which produced circulating concentrations consistent with human exposures, did not act as an aromatase inhibitor; rather, dietary intake of genistein negated the inhibitory effect of an aromatase inhibitor letrozole (a 3^rd ^generation aromatase inhibitor), by stimulating the growth of aromatase-expressing estrogen-dependent breast tumors [106]. This study raises concerns about the consumption of genistein-containing products by postmenopausal women with advanced breast cancer who may be treated with letrozole. Genistein suppressed promoter I.3/II transactivity in SK-BR-3 cells (an ER-negative breast cancer cell line), however, in HepG2 cells, genistein was found to induce promoter-specific aromatase mRNA expression with significant increases in promoters I.3 and II [89]. In addition, the phosphorylated forms of PKCα, p38, MEK and ERK1/2 kinases were also induced in HepG2 cells by genistein [89]. There are also some reports of a weak inhibition of aromatase enzyme activity by genistein as well [80,107] and a decrease in the transcription of *Cyp19 *mRNA in human granulosa luteal cells [108].

Quercetin is one of the most abundant flavonols found in plants. Quercetin was found to inhibit human aromatase activity in placental microsomes [109]. When tested in cellular systems utilizing adrenocortical carcinoma cells, preadipocyte cells, or in co-culture experiments, it exhibited either a mild or no effect [86,110,111]. In the primary culture of human granulosa-luteal cells quercetin was able to reduce aromatase mRNA expression in a dose-dependent manner after an exposure period of 48 h [108]. In another study, H295R human adrenocortical carcinoma cells were exposed to quercetin for 24 h and an increase in aromatase enzyme activity was observed at lower concentration, while a decrease in the enzyme activity was observed at higher concentrations [105]. Quercetin increased p II and I.3-specific aromatase transcripts about 2.6-and 2-fold in H295R cells after 24 h exposure probably by enhancing intracellular cAMP levels [105].

Isoliquiritigenin, a flavonoid from licorice (*Glycyrrhiza glabra*), was found to be an inhibitor of aromatase enzyme activity in vitro [90]. Moreover, this compound was able to block MCF-7aro cells(MCF-7 cells stably transfected with *CYP19*) growth and when added in diet inhibited significantly the xenograft growth in ovariectomized athymic mice transplanted with MCF-7aro cells [90]. Isoliquiritigenin also inhibited aromatase mRNA expression and suppressed the activity of *CYP19 *promoters I.3 and II [90] in MCF-7 cells. Furthermore, binding of C/EBP to PII promoter of *CYP19 *was suppressed by isoliquiritigenin [90]. This study indicated that isoliquirititigenin has the potential to be used as a tissue-specific aromatase inhibitor in breast cancer.

The aromatase inhibitory activity of grapes and grape seed extracts (GSE) has been studied by many investigators [61,83,91]. The active chemicals found in grapes and red wine are procyanidin dimers that are also present in high concentrations in grape seeds [87]. GSE is composed of about 74-78% of proanthocyanidins and <6% of free flavanol monomers such as catechin, epicatechin, and their gallic acid esters [87]. Through the suppression of the expression of CREB-1 and glucocorticoid receptor (GR), grape seed extracts (GSE) has been found to decrease the expression of aromatase in MCF-7 and SK-BR-3 cells by suppressing the activity of promoters I.3/II, and I.4 in a dose-dependent manner [87]. The GSE (IH636) is in phase I clinical trials for the prevention of breast cancer in postmenopausal women who have an increased risk of breast cancer development [61].

The grape peel contains resveratrol, a polyphenolic compound which has structural similarity with estrogen [91]. This nonflavonoid phytoestrogen inhibited aromatase activity in MCF-7aro cells. In SK-BR-3 cells resveratrol significantly reduced aromatase mRNA and protein expression in a dose-dependent manner [91]. Moreover, this compound was able to repress the transactivation of *CYP19 *promoters I.3 and II in SK-BR-3 cells [91], which indicate that resveratrol could be able to reduce localized estrogen production in breast cancer cells.

## Future directions

The expected direct outcome of aromatase inhibition is the maintenance of low levels of estrogen in the breast and surrounding adipose tissue. Understanding the molecular mechanism by which aromatase promoters I.4 and I.3/II are regulated is clinically significant and useful for developing new drugs. Although only a few plant products have been documented to mediate their effects through aromatase promoters, there are many more potent natural products (such as white button mushroom (*Agaricus bisporus*) which is in phase I trials [83]) which could be potential candidates for future study. Moreover, accumulating evidence suggests that beside transcription factors and co-regulators there are many other factors such as cyclooxygenases (COX) which are involved in tissue-specific aromatase promoter regulation [112,113]. Selective COX inhibitors from natural products can be used to suppress *CYP19A1 *gene expression. Studies also indicate that *CYP 19A1 *regulations are also under epigenetic control, including DNA methylation, and histone modification, which can add a new layer of complexity in the regulation of the aromatase gene [114]. DNA methylation generally occurs in gene promoters where the CpG rich dinucleotides are located. However, DNA methylation of CpG-poor promoter regions has also been shown as a mechanism of mediating tissue-specific gene transcription through the inhibition of transcription factor binding [115,116]. Aromatase promoter I.3/II has six CpG dinucleotides subjected to methylation of cytosines and can be considered as CpG-poor promoter. However, in human skin fibroblasts hypermethylation of almost all six CpG sites resulted in markedly reduced aromatase promoter I.3/II activity, whereas hypomethylation of only two of the six sites led to increased promoter activity associated with an increase in cAMP [14]. In contrast to these studies, in breast adipose fibroblasts (BAF) promoter I.4 and I.3/II derived mRNA were not dependent on the CpG methylation status within respective aromatase promoters [114]. Further, DNA methylation is catalyzed by DNA methyl transferases (DNMTs). Inhibition of DNA methylation by 5-aza-2'-deoxycytidine, which is also a specific DNMT inhibitor, increased *CYP19 *mRNA expression in BAFs and breast cell lines [114]. These studies indicate that disruption in epigenetic regulation may give rise to increase in aromatase levels in the breast [114]. There are many synthetic chemicals that are undergoing clinical trials to be used as epigenetic drugs (epidrugs) for breast cancer treatment [117]. The major problems of these drugs are the unwanted side effects. Many natural products have the potential to be used as better epidrugs than synthetic epidrugs. One of the best examples is (-) - epigallocatechin-3-gallate from green tea which is used as demethylating agents for breast cancer patients [118-120]. Therefore extensive investigations in natural products seem promising or necessary.

## Conclusions

Aromatase is a well-established molecular target and the AIs are proving to be an effective new class of agent for the chemoprevention of breast cancer. Regulation of aromatase expression in human tissues is a complex phenomenon, involving alternative promoter sites that provide tissue specific control. The promoters I.3 and II are the major promoters directing aromatase expression in breast cancer. The drugs that can selectively inhibit aromatase expression may be useful to obviate side effects induced by the nonselective AIs. Although many synthetic chemicals are used to inhibit tissue-specific inactivation of aromatase promoters I.3 and II, in the literature only a few natural products (we have included six of them) have been reported with such activities. More studies on natural products are necessary to find an appropriate tissue-specific AI.

## List of abbreviations used

AIs: Aromatase inhibitors; COX: Cyclooxygenase; E1: estrone; E2: 17β- estradiol; ER: Estrogen receptor; PGE: prostaglandin; PPAR: Peroxisome proliferator activator receptor; C/EBP: CCAT/enhancer binding protein.

## Competing interests

The authors declare that they have no competing interests.

## Authors' contributions

SIK, JZ, IAK, LAW and AKD are contributed in literature review, graphics work and writing the manuscript. All authors read and approved the final manuscript.

## Author's information

Shabana I. Khan is the Senior Scientist at the National Center for Natural Products Research and Associate Professor of the Department of Pharmacognosy at the University of Mississippi, University, MS 38677, USA. Jianping Zhao is the Associate Research Scientist at the National Center for Natural Products Research at the University of Mississippi, University, MS 38677, USA. Ikhlas A. Khan is the Assistant Director of the National Center for Natural Products Research and Professor of Pharmacognosy, School of Pharmacy of the University of Mississippi, University, MS 38677, USA. Larry A. Walker is the Director of the National Center for Natural Products Research at the University of Mississippi, and Associate Director for Basic Research Oxford, University of Mississippi Cancer Institute and the Professor of Pharmacology, School of Pharmacy of the University of Mississippi, University, MS 38677, USA, Asok K. Dasmahapatra is the Research Scientist at the National Center for Natural Products Research and Assistant Professor of the Department of Pharmacology, School of Pharmacy of the University of Mississippi, University, MS 38677, USA.
